# Successful pregnancy outcome after fresh embryo transfer in a dual stimulation cycle - a case report

**DOI:** 10.5935/1518-0557.20220033

**Published:** 2023

**Authors:** Chithira Pulimoottil Vignarajan, Neena Malhotra

**Affiliations:** 1 ART center, Department of Obstetrics and Gynecology, All India Institute of Medical Sciences, New Delhi-110029, India

**Keywords:** premature LH surge, dual stimulation, fresh embryo transfer

## Abstract

Controlled ovarian stimulation by antagonist protocol sometimes presents unpleasant surprises in the form of unexpected premature rupture of follicles despite well-timed daily administration of the antagonist. In such cases ovum pick up cannot be done, dual stimulation of the next crop of follicles may be pursued to salvage the cycle. A ‘freeze all’ strategy is usually implemented in all cases of dual stimulation because of embryo-endometrial asynchrony. Here we present a case where dual stimulation was followed by fresh embryo transfer with a successful pregnancy outcome.

## INTRODUCTION

Antagonists are routinely used in the control of premature Luteinizing Hormone (LH) surge during controlled ovarian stimulation (COS) in In vitro fertilization (IVF) cycles. Antagonist cycle offers the advantages of prompt control of LH surge, shorter duration of stimulation, decreased gonadotropin requirement and lower costs, significantly reduced risk of Ovarian Hyper Stimulation Syndrome (OHSS) with the possibility of agonist trigger in expected high responders, and hence more patient-friendly (Copperman & Benadiva, 2013). However, they might fail to efficiently control the premature LH surge in a subset of patients despite proper timing and dosage of antagonist administration and maintenance of cold chain of the antagonist drug. If these LH upsurges are substantial and ensue with premature progesterone (P) rises, ovulation becomes impending (Frattarelli *et al*., 2013). In such situations, stimulation of the next follicular cohort may be embarked upon to salvage the cycle. So-called dual stimulation is habitually combined with the ‘freeze all’ strategy because of embryo-endometrial asynchrony. However, in some unfortunate circumstances, embryos may be of very poor quality and consequently may not be amenable to the freezing process. A fresh transfer may be a feasible option in such situations. Here we present the case of a 30-year-old female who had premature LH surge during COS and hence underwent dual stimulation followed by fresh transfer, which resulted in a live birth.

## CASE DESCRIPTION

A 30-year-old female with PCOS presented with a 6-year primary infertility. Her BMI was 20.5 kg/m^2^, antral follicle count 30 and serum antimullerian hormone 15 ng/ml. Serum LH on day 2 of the periods was 9.8 mIU/mL. Her husband had obstructive azoospermia. IVF was undertaken in view of PCOS with male factor requiring surgical sperm retrieval. COS by antagonist protocol was started from day 2 of periods with 200 IU of recombinant FSH daily. Antagonist (Cetrorelix 0.25 mg/day S/C) was started from day 6 of stimulation when the lead follicle measured 14 mm. On day 8 of the stimulation, she had 5 follicles measuring 14 mm and 6 follicles measuring 13 mm. The subsequent ultrasound, on day 9 of stimulation showed no follicle ˃ 14 mm. All the follicles were found to be ruptured with free fluid in the pelvis and we noticed echogenicity inside the follicles, suggestive of hemorrhage. Serum estradiol and progesterone on day 9 of stimulation were 321 pg/ml and 28 ng/ml, respectively. Gonadotropin injections were stopped. The couple was explained the unanticipated rupture of follicles and the chances of poor oocyte retrieval. They were given the option of continuing the same cycle by applying a dual stimulation protocol and were explained the ambiguity of the outcome.

The couple opted to continue with the cycle by dual stimulation. The patient was administered only the antagonist injection for the next 5 days (Cetrorelix 0.25 mg/day S/C). She was started on gonadotropin injections at a higher dose of gonadotropins (300 IU r-FSH and 75 IU hMG daily) given the high progesterone levels and the ensuing chance of resistance to stimulation. After 2 days of stimulation, she menstruated. On day 6 of stimulation, the serum estradiol level was 693 pg/ml and the antagonist was started. On day 8 of stimulation, there was a single follicle measuring 15 mm, six follicles measuring 14 mm and her serum estradiol was 698 pg/ml. On day 9 of stimulation, the ultrasound findings were the same with a serum estradiol level of 695 pg/ml and serum progesterone of 1.07 ng/ml. The endometrial thickness was 7.6 mm, triple line pattern. The trigger was administered with 250 µg of recombinant hCG because of follicle growth stagnation and plateauing of the serum estradiol levels. Four grade ii oocytes were retrieved. The husband’s sperms were retrieved by percutaneous epididymal sperm aspiration. The oocytes were subjected to intracytoplasmic sperm injection (ICSI), taking the chances of fertilization failure, which might happen because of poor oocyte and sperm quality. The fertilization check was done 16 hours post-ICSI. Two oocytes were fertilized on day 1. The following day, two day-2 embryos (4 celled grade C) were transferred to the uterus. Luteal support was given with micronized progesterone (Inj. Susten 100 mg IM daily).

Serum β hCG on day 16 of embryo transfer was 846 IU/L. A single intrauterine fetus with cardiac activity was documented 2 weeks following positive serum β hCG. The antenatal period was uneventful. At 39 weeks of gestation, she came in spontaneous labor and delivered a 2.8 kg healthy male baby.

## DISCUSSION

Ovarian stimulation with exogenous gonadotropins for multi follicular development during an IVF cycle is inevitably associated with supraphysiological levels of serum estradiol and hence the risk of premature LH surge. Elevated LH levels in the follicular phase affect oocyte quality by causing early resumption of meiosis and premature oocyte maturation and ovulation, causing either lower implantation or increased miscarriage rates (Loumaye *et al*., 1989)and to evaluate a putative impact of the flare-up effect on follicular recruitment and subsequent IVF. Eighteen highly selected patients were randomely divided in two groups. Nine patients received a short-term administration of Buserelin (Hoechst, AG, Franfurt/Main, FRG. Elevated follicular LH levels have a detrimental effect on pregnancy rates in IVF cycles (Tarlatzis *et al*., 1995)20 cycles. Hence the addition of some sort of GnRH analogue for blocking the premature LH surge is an integral part of COS cycles.

GnRH-antagonists compete with the endogenous GnRH for the pituitary GnRH receptors, effecting a rapid suppression of pituitary secretion of both FSH and LH without a flare effect. Antagonist administration in the late follicular phase prevents premature LH surge and premature luteinization (Ozelci *et al*., 2019).

However, premature LH rise, which is defined as LH ≥10 mIU/ml, still happens in around 7% of cases despite antagonist administration (Allegra *et al*., 2007). When the premature LH rise is combined with premature progesterone rise ≥2 ng/ml, it is known as premature luteinization, and this phenomenon is reported in about 1-2% of cases despite proper administration of antagonist (Allegra *et al*., 2007; Bakas *et al*., 2011; Ozelci *et al*., 2019). One study has reported premature luteinization of even 9.5% despite the antagonist (Ertunc *et al*., 2010).

Why this phenomenon happens despite the proper timing, dosage and route of antagonist administration and maintenance of the cold chain is not clear. This could be probably due to amplified endogenous GnRH release in response to escalating serum estradiol concentrations (Frattarelli *et al*., 2013).

Some studies have suggested the likely possibility of the pituitary remaining unprotected against the stimulating effect of estradiol (E_2_) between the two injections, 24 hours apart, which stimulate intracellular mechanisms that result in LH release. The authors postulated that even though after the first injection basal LH levels were markedly suppressed, normal pulsatility and pretreatment LH levels were reestablished at least 6 hours before the second injection, reflecting a decrease in the antagonist levels towards the end of 24 hours after administration (Griesinger *et al*., 2006). Any of these factors would have played a role in the premature LH surge seen in our patient.

This is a very perplexing state for the patient as well as the reproductive medicine specialists’ treating them. In such situations, dual stimulation of the upcoming cohort of follicles may be tried as a rescue strategy to salvage the cycle and help reducing the patient’s agony on cycle cancellation. Dual stim is usually embarked in situations like poor responders for oocyte and embryo pooling to improve the possibilities of euploid blastocyst transfer (Vaiarelli *et al*., 2018). But our experience has shown that dual stim could be of help in desperate situations like the present case scenario.

Studies have shown premature action of LH on the follicular granulosa cells in PCOS patients (Jonard & Dewailly, 2004). Excessive mRNA expression of the LH receptor on the granulosa cells of PCOS patients has also been reported (Jakimiuk *et al*., 2001). These factors could account for the stagnation and arrest of follicular growth seen in our study.

Dual stimulation combined with the freeze-all strategy is the usual dictum (Pirtea *et al*., 2019). The novelty of the present case report lies in the fact that ‘fresh transfer’ was performed following dual stimulation. This was based on a situation of poor-quality embryos and was made possible by administering the antagonist for five days between the two stimulations, causing the estradiol levels from the first cohort to fall and menstruation to ensue, followed by regeneration of the endometrium by estradiol from the second cohort of follicles. Our experience also suggests that dual stimulation of the second cohort of follicles can happen in situations where oocytes could not be retrieved from the first cohort of follicles.
